# GabROP: Gabor Wavelets-Based CAD for Retinopathy of Prematurity Diagnosis via Convolutional Neural Networks

**DOI:** 10.3390/diagnostics13020171

**Published:** 2023-01-04

**Authors:** Omneya Attallah

**Affiliations:** Department of Electronics and Communications Engineering, College of Engineering and Technology, Arab Academy for Science, Technology and Maritime Transport, Alexandria 1029, Egypt; o.attallah@aast.edu

**Keywords:** retinopathy of prematurity (ROP), deep learning, computer assisted diagnosis (CAD), artificial intelligence (AI), eye disease, Gabor wavelets (GW)

## Abstract

One of the most serious and dangerous ocular problems in premature infants is retinopathy of prematurity (ROP), a proliferative vascular disease. Ophthalmologists can use automatic computer-assisted diagnostic (CAD) tools to help them make a safe, accurate, and low-cost diagnosis of ROP. All previous CAD tools for ROP diagnosis use the original fundus images. Unfortunately, learning the discriminative representation from ROP-related fundus images is difficult. Textural analysis techniques, such as Gabor wavelets (GW), can demonstrate significant texture information that can help artificial intelligence (AI) based models to improve diagnostic accuracy. In this paper, an effective and automated CAD tool, namely GabROP, based on GW and multiple deep learning (DL) models is proposed. Initially, GabROP analyzes fundus images using GW and generates several sets of GW images. Next, these sets of images are used to train three convolutional neural networks (CNNs) models independently. Additionally, the actual fundus pictures are used to build these networks. Using the discrete wavelet transform (DWT), texture features retrieved from every CNN trained with various sets of GW images are combined to create a textural-spectral-temporal demonstration. Afterward, for each CNN, these features are concatenated with spatial deep features obtained from the original fundus images. Finally, the previous concatenated features of all three CNN are incorporated using the discrete cosine transform (DCT) to lessen the size of features caused by the fusion process. The outcomes of GabROP show that it is accurate and efficient for ophthalmologists. Additionally, the effectiveness of GabROP is compared to recently developed ROP diagnostic techniques. Due to GabROP’s superior performance compared to competing tools, ophthalmologists may be able to identify ROP more reliably and precisely, which could result in a reduction in diagnostic effort and examination time.

## 1. Introduction

Retinopathy of prematurity (ROP), a vasoproliferative condition, affects premature babies that weigh less and have shorter gestational times [1]. ROP, which disproportionately affects low-and middle-income nations, is one of the main causes of childhood blindness globally. ROP has also become a concern that cannot be disregarded in both developed and developing countries, particularly in developing countries, as the survival rate of preterm children has increased globally. For instance, it is roughly predicted that approximately 20,000 preterm newborns in China, where there are about 2 million premature births each year, have ROP [2]. Additionally, ROP is thought to cause blindness or serious vision impairment in roughly 30,000 preterm infants each year globally [3]. However, eye exams and the availability of therapy for those at risk can almost avoid blindness from ROP.

There are a number of obstacles to ROP screening and diagnosis in developing nations. Equipment for medical imaging and screening ROP patients is insufficient. Furthermore, there are not a lot of staff available to acquire retinal scans for ROP. Additionally, there are not many skilled ophthalmologists, and the training process for them is inconsistent. Furthermore, developing nations do not adequately implement the ROP screening strategy. As a result, the lack of early detection and prompt treatment causes a large number of preterm newborns to go blind in developing nations [4]. Through timely screening, the advancement of ROP can be observed. Traditionally, manual screening is conducted by ophthalmologists. However, it takes a lot of time, is costly, and depends on the individual ROP expert’s assessment. Therefore, automated systems are essential to facilitate diagnosis. Currently, ophthalmologists employ the RetCam imaging system to capture fundus images of preterm newborns for the prevention and treatment of ROP instead of the conventional binocular indirect ophthalmoscopy (BIO). Retcam technology has gained popularity for its straightforward operation, wide-angle imaging, and excellent resolution. Multiple-angle fundus photographs can be recorded, saved, and taken with the Retcam scheme. Additionally, it is better for scientific study, education, and medical monitoring and inspection. All of these advantages demonstrate that it can identify ROP when used in conjunction with digital image analysis techniques [5].

Over the past ten years, automated computer-aided diagnostic (CAD) systems for disease identification have become increasingly popular using artificial intelligence (AI) [6,7,8,9,10,11]. More notably deep learning (DL) has been extensively used in the medical domain [12,13,14,15,16,17,18]. DL approaches are preferred over conventional machine learning methods as they do not require any processing of the data such as segmentation and feature extraction [19]. In several medical research applications, convolutional neural networks (CNNs), a subset of DL approaches, have shown clinician-level effectiveness in pattern recognition and analyzing medical images [20,21]. Automatic screening methods based on AI could save time, help to reduce this subjectivity, and aid to reduce the diagnostic discrepancy between specialists [19]. Digital fundus photography is becoming more widely available, and thus there has been a lot of work done on using AI specially CNNs to give healthcare providers more access to patients with ROP and to increase interobserver agreement by providing an objective and quantitative framework for patient evaluation [22,23,24]. Despite the fact that this would be advantageous for any ROP screening population, it might have the most effects in low- and middle-income nations where the disease frequency is the highest and the human resource pool is the smallest [23].

A well-known technique that is frequently used to examine medical photos is texture analysis. Multiple calculation stages are used for medical images as part of the textural analysis [25]. The accurate identification of numerous diseases has been made possible by the combination of texture analysis and AI techniques [26]. Texture analysis’s key advantage comes from its capacity to extract textural information from medical images that represent disease or tumor patterns. This information might help the AI tools to correctly diagnose the disease [27]. One of the most extensively utilized textural analysis techniques is Gabor-wavelets (GW) which is employed in a variety of medical applications [28]. This paper presents GabROP, a robust and efficient diagnostic tool based on ensemble DL techniques and the GW texture analysis method, to accurately and automatically diagnose ROP in its early stages. Rather than employing only the original fundus images to perform the diagnostic procedure, GabROP utilizes textural images generated from the GW approach as well to learn the DL models. With GabROP, ophthalmologists may be able to diagnose ROP promptly and correctly. Additionally, this article aims to create a CAD tool that can distinguish photos with and without ROP. By using GabROP, the diagnosis procedure can be less time- and labor-intensive.

The following are the study’s major contributions:

1. Utilizing the GW texture analysis method to analyze fundus images and produce multiple sets of GW images to train DL models along with the original fundus images.
2. Three cutting-edge pre-trained CNN models with various architectures are employed to construct GabROP.
3. Integrating several textural features attained through training each CNN with a different set of GW images using DWT, resulting in textural-spectral-temporal interpretation. Moreover, a reduction in the dimension of integrated features is performed.
4. Merging spatial information obtained using features extracted from each CNN constructed with the actual fundus photos with the textural-spectral-temporal representation of the combined features acquired via the same CNN constructed with the multiple sets of GW images.
5. Incorporating fused features of the three CNNs, thus combining the benefits of their distinct structures.

The organization of this study is as follows. Section 2 provides examples of earlier CAD tools for ROP diagnosis. The methods and resources are presented in Section 3. Additionally, GabROP, the suggested CAD tool, is introduced with a description. Section 4 provides information on the evaluation criteria and parameter adjustment. The GabROP results are presented in Section 5. The key findings of GabROP are discussed in Section 6 along with limitations and future directions. The paper is concluded in Section 7.

## 2. Related CAD Systems for ROP Diagnosis

Throughout the past few decades, AI-based CAD tools have demonstrated exceptional performance in analyzing medical images. Inspired by this success, several AI-based CAD tools have been adopted for ROP diagnosis and have shown encouraging results. The selection criteria for the relevant publications are based on recent research that was solely concerned with identifying ROP, i.e., the research created CAD systems for diagnosing diabetic retinopathy in exclusively preterm newborns. Several studies used standard machine learning methods to diagnose ROP, and among them the study [29] used 87 fundus images to identify non-plus ROP, pre-plus, plus, and illness via a variety of machine learning classifiers. The authors achieved an 80.15% accuracy. On the other hand, using 110 pictures of 41 patients, the authors of the studies [30,31] used filters of Gabor to discriminate amongst ROP disease plus/non-plus. Whereas to distinguish between pre-plus and plus ROP disorders, 20 fundus photos were used to create an ROP tool in the study [32]. Similarly, a diagnostic technique named “i-ROP” was proposed in [33] to identify pre-plus, healthy, and plus ROP illness via a support vector machine (SVM) classifier reaching 95% accuracy (with 77 pictures). The classification models for the earlier tools were built using a few, low-quality photos. These tools need to manually extract vessel features and segment the vessels, which can decrease the accuracy of diagnosis due to errors and professional biases that might happen while selecting the target vessels. Additionally, image processing tasks, e.g., segmentation and feature extraction, need a lot of time. This makes more automated, dependable technologies, including those based on DL methods, necessary.

Numerous CAD tools for ROP diagnosis based on DL methods have recently been released. These tools made use of transfer learning (TL) [34], which reuses previously learned convolutional neural networks (CNNs) on a different classification task with fewer photos, like the one at hand, but with enormous datasets, such as ImageNet. TL has shown its ability to improve diagnostic accuracy in several medical domains [35,36]. Therefore, it is employed in constructing CAD tools for ROP diagnosis. The paper [3] proposed a novel attention-aware and deep supervision-based network (ADS-Net) to detect the presence of ROP (Normal or ROP) and 3-level ROP grading reaching an accuracy of 96.16%. Likewise, a “DeepROP” automated system, which is based on DL, was introduced [37]. 11707 pictures were used by the authors. Images were first classified by the algorithm as normal or ROP, and then ROP images were diagnosed as severe or minor. The system classified normal from ROP with a 96.64% sensitivity and 99.33% specificity, and severe from minor with an 88.46% sensitivity and 92.31% specificity. Meanwhile, the study [38] utilized U-Net and DenseNet CNNs to distinguish normal fundus images from ROP images of different severity using 7939 images. Similar to this, the “i-ROP” system was created in [39] and is built by two CNNs, the first of which is used for segmenting images and the second of which is used to identify healthy, pre-plus, and plus ROP conditions. A 96.5% sensitivity and 94% specificity using 5511 retinal images were the result of the investigation. Moreover, the authors of [40] introduced the “I-ROP ASSIST” pipeline to distinguish between normal and plus ROP pathologies. The authors used U-Net CNN to segment the photos, and then took these segmented images and extracted manually created features to train numerous machine learning classifiers. The highest accuracy reached was 94%.

On the other hand, the article [41] [] developed a method that combined multiple instance learning (MIL) with deep learning (DL), in which images were separated into identical regions, and features were then retrieved using a CNN from these patches. To differentiate between normal and ROP cases, variables of the same image are merged. The system’s results for diagnostic accuracy were 83.33%, 100% sensitivity and specificity, and 71.43%, respectively. To identify normal and plus ROP disease, “ROP.AI,” an automated tool, was developed by the authors of the study [42]. The classification model had a 98.0% specificity rate and a 96.6% sensitivity rate. In contrast, a modified version of ResNet-50 was used by the study [43] to identify ROP, where an attention module and channel were added to the conventional ResNet-50 structure. Similar to this, the authors of the study [44] built their model using various ResNet iterations. Alternatively, few other studies used several CNNs to construct their models. Among them, to diagnose ROP in preterm infants, the study [45] introduced a framework based on several CNNs. The authors first made a distinction between healthy and ROP instances before categorizing the severity of ROP into moderate and severe. The authors demonstrated that VGG-16 had the best output by comparing the output of the various CNNs. The VGG-16 achieved 96% accuracy, 96.6% sensitivity, and 95.2% specificity for normal versus ROP. The VGG-16 model achieved 98.82% accuracy, 100% sensitivity, and 98.41% specificity for severity classification. Later, DIAROP, an automatic and efficient diagnostic tool, was presented in [46]. By first extracting spatial data from the four CNNs using TL and then utilizing the fast Walsh Hadamard transform (FWHT) on these spatial features to create spectral features. These features were fused using several methods. Fused features were then fed into three classifiers reaching the highest accuracy of 93.2%. Additionally, in [47], a framework was implemented via three CNNs. ROP cases were originally identified by the model, which then divided their severity into moderate and severe cases. Using the Inception CNN, a 97% and 84% maximum accuracy for healthy/ROP and moderate/severe was attained. “DeepDR,” a different automated approach based on DL, was suggested in [48]. The system used a group of CNNs to first diagnose ROP and then to determine the severity of it. The two fusion steps of DeepDR are called feature-based fusion and probability-based fusion, respectively. The findings demonstrated that the peak performance for recognizing ROP was obtained by merging the average probability of Xception, Inception, and InceptionResNet CNNs. The identical CNNs attained a sensitivity of 98.1% and a specificity of 98.9% when determining the severity level.

The outcomes of DL-based diagnostic tools have demonstrated that these technologies are equivalent to professionals in ROP disease diagnosis and can even do it more accurately. This means that they could be used to diagnose ROP [5]. They do have some limitations, though. For example, the majority of earlier techniques relied solely on spatial DL features, whereas combining this category of features among other feature categories, like spectral-temporal features, can improve the accuracy of image classification [49,50]. Additionally, practically most of them only used one CNN to do classification or extract features; nevertheless, it has been demonstrated that combining the features of many CNNs can improve classification performance [51,52,53,54]. Furthermore, all of the previous studies mainly depend on using the original fundus images to train the DL models, however, combining features obtained from textural-based images with the original fundus may improve the accuracy. Therefore, in this paper, an efficient CAD tool called GabROP, based on multiple CNNs, and the Gabor wavelet (GW) textural analysis technique are proposed. GW method is employed to analyze fundus images. Textural images generated using GW are used along with the original fundus images to train the ensemble of CNNs. GabROP consists of three fusion stages. Initially, in the first fusion stage, textural features obtained from the CNNs learned with textural GW sets of images are fused using discrete-wavelet transform (DWT) which provides spectral-temporal information. Then, in the second fusion stage, spatial features extracted from each CNN trained with the original images are concatenated with textural-spectral-temporal features obtained via the CNNs built with GW texture images. Last but not least, discrete cosine transform (DCT) is employed to further merge the three CNNs’ combined features.

A comparative problem regarding Age-related macular degeneration (AMD) was illustrated in the challenge called “The Automatic Detection challenge on Age-related Macular degeneration” (ADAM) [55]. AMD is the foremost reason for blindness in elder people worldwide. The most economical imaging technology for detecting retinal disorders is the color fundus modality. ADAM challenge offered a dataset for AMD detection consisting of annotated fundus images. The main goal of the challenge was to develop DL models for segmenting lesions and optic discs, and then the classification of images to AMD and non-AMD. One additional task was to locate Fovea. Eleven teams finally participated in the challenge. These teams used several DL models to perform the classification task including EfficientNet versions, DenseNet versions, Inception-ResNet, ResNeXt, SeNet, Xception, Inception, ResNet-50, Autoencoders with ResNet-50, ResNet-101. Out of the eleven teams, five utilized ensemble DL models of different structures or the same architecture but various setups. The peak area under receiving operating characteristics curve (AUC) of 0.9714 was attained by the VUNO EYE TEAM. On the other hand, for the detection and segmentation tasks of the optic disc, other combinations of DL models were employed, such as fully convolutional network (FCN) with ResNet-50, FCN with VGG-16, U-Net and DeepLab with ResNet, FCN with EfficientNet, U-Net with EfficientNet of several versions, U-Net with several versions of ResNets, U-Net with Inception, U-Net with DenseNet-101. The maximum dice coefficient of 0.9486 was achieved by the XxlzT team. For fovea localization, multiple FPNDL models were applied, such as U-Net with EfficientNet, three feature pyramid networks (FPNs) with EfficientNet and ResNet, VGG-19, and Inception CNNs, Nested U-Net, FCN with ResNet-50, GAN, two U-Nets with Mask regional CNN (RCNN) and ResNet, EfficientNet of several versions, Yolo-v2, and faster RCNN. The highest Euclidean distance of 18.55 was attained by the VUNO EYE TEAM. Finally, for the segmentation and detection task of lesions, U-Net with EfficientNet, U-Net- with Residual blocks, DeepLab-v3 with ResNet or Xception, FPN with EfficientNet, Nested U-Net+ FPN + DeepLab-v3 with EfficientNet, FCN with VGG-16, U-Net with inception, EfficientNet, ResNet-50, and DenseNet-10, U-Net with EfficientNet and ResNet-50, and DeepLab-v3. The VUNO EYE TEAM also achieved the first rank for this task.

The proposed approach differs from the methods employed in the ADAM challenge in the following two aspects. First, it did not use the fundus images directly to train the DL models. Alternatively, in GabROP, the GW method is used to analyze color fundus photos. Textural images produced by GW are used to train the ensemble of CNNs alongside the original fundus images. This is because blending textural-based image features with genuine fundus photos will probably enhance diagnostic accuracy. Second, GabROP did not utilize any segmentation or detection-based-DL models for ROP.

## 3. Materials and Methods

### 3.1. ROP Dataset Description

**Figure 1:**
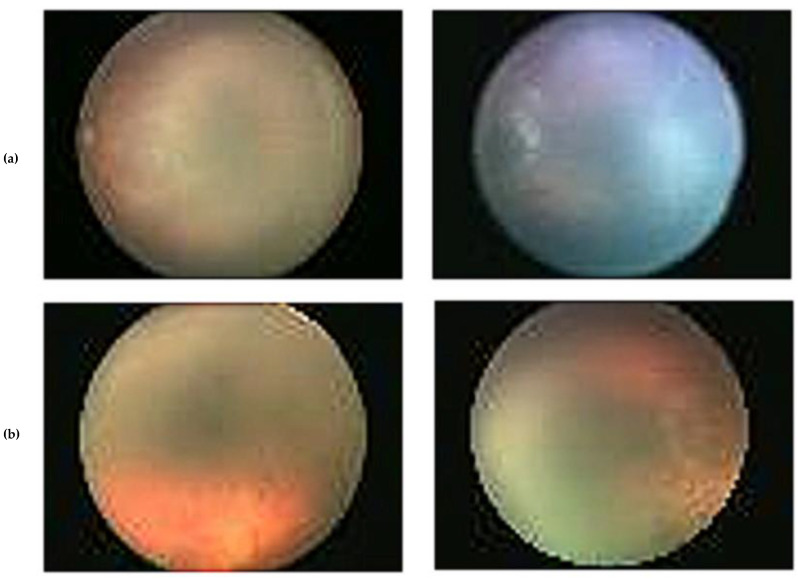
Examples of the dataset’s images, (**a**) diseased and (**b**), not diseased.

The 30 hospitals that make up the ROP Collaboration Group (RCG), which is dispersed throughout China, were in charge of gathering the ROP pictures. Since it provides ROP screening services to all other partner hospitals, Shenzhen Eye Hospital (SEH) served as the initiative’s driving force. Similar ROP screening criteria were used by all of these institutions to choose the cases that would be included in the dataset collection. In 2018, the ROP screening data included 26,424 newborns and 420,365 photos. After the RCG’s screening procedures, more than 37 thousand unqualified images were removed from the original 420,365 images. Images that are highly blurred, images that are very dark or very bright, and photographs that are not fundus are considered as unqualified. The inclusion criteria for participating in the data acquisition procedure are as follows: First, they ought to be under 2000 g in weight at birth. Second, 2000 g premature neonates who have significant systemic diseases at birth. There were about 890 newborns who qualified. An experienced technician used Retcam 2 or Retcam 3 to capture the photos. The images obtained throughout the screening method were separately annotated by five pediatric ophthalmologists as “Disease” and “Not Diseased.” Among these pediatric ophthalmologists, two were chief physicians with experience over 20 years in ROP diagnosis and treatment, two were attending physicians with experience over 10 years, and one was a resident with experience over 3 years. After discussion among the panel of ophthalmologists regarding inconsistencies, a conclusion was made. The reference [56] provides additional information on the data-collecting procedure. Images from the dataset are displayed as examples in Figure 1. Since the data collection did not involve any clinical information or sensitive patient information, the RCG review board has approved its use without approval and registration. Each premature infant’s eye was imaged from ten different angles during each screening procedure resulting in 20 images for both eyes of every neonate. For training set (set 1), 8090 pictures were gathered for ROP cases, compared to 9711 for healthy eyes. While for set 2, 155 images were available for ROP and 1587 images for healthy eyes. The dataset did not categorize images of different stages/severities of ROP. Table 1 describes the characteristics of the subjects.

### 3.2. Proposed GabROP Tool

**Figure 2:**
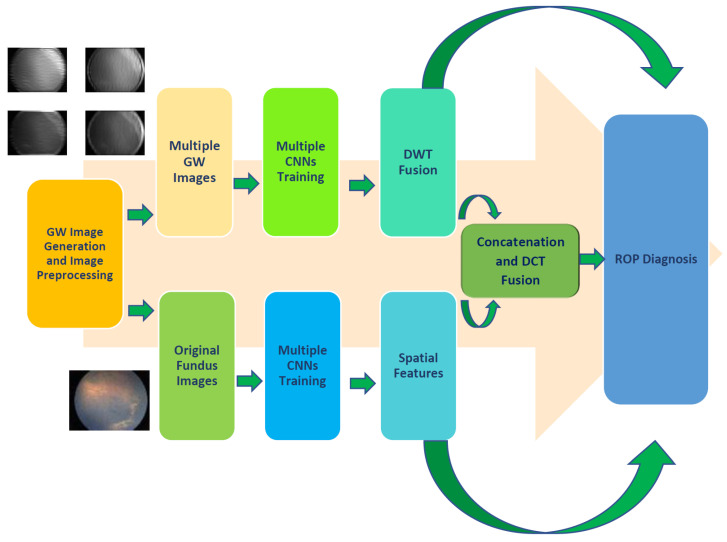
The stages of the proposed GabROP CAD tool.

For the early detection of ROP, this study proposes an effective CAD tool called GabROP that relies on multiple CNNs, and GW textural analysis techniques. GabROP is divided into four stages: GW image generation and preprocessing, multiple CNN training and extraction of features, triple fusion, and diagnosis stages. Initially, fundus photos for preterm infants that were obtained with Retcam are used to train three DL models. Furthermore, these fundus images are analyzed with the GW approach. Afterward, images generated using GW are used as well to train these DL models after being augmented and resized. Next, CNNs trained on both types of images (original fundus images and GW images) are used to extract deep features. The feature fusion phase involves three fusion stages. First, for each CNN, texture-deep features obtained from each CNN trained with GW images are combined using DWT. After that, textural information obtained from the CNNs trained with GW photos is merged with spatial information obtained utilizing deep features retrieved by CNNs built with the actual fundus images. DCT is then utilized to combine both kinds of deep features that were mined from the three CNNs. Finally, in order to conduct classification, three classifiers are used, including linear discriminate analysis (LDA), support vector machine (SVM), and ensemble discriminate classifier (ESD). The stages of the proposed GabROP tool are shown in Figure 2.

#### 3.2.1. Gabor Wavelets Image Generation and Preprocessing

**Figure 3:**
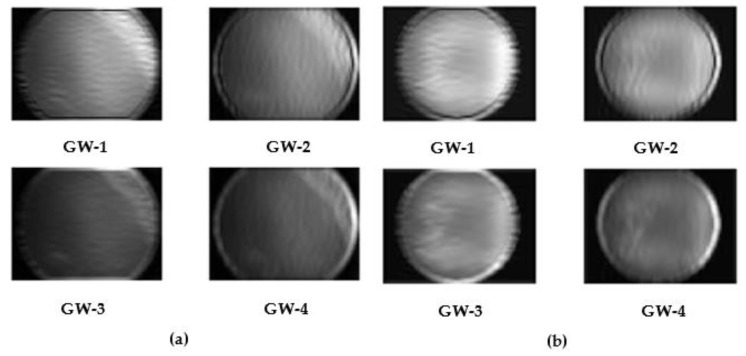
Samples of the generated GW images for both classes of the ROP dataset, (**a**) Diseased, (**b**) Not Diseased.

One of the best methods for determining the frequency response of audio, picture, and video signals is the Fourier transform. However, the Fourier transform loses the frequency spectrum information, making it unsuitable for picture restoration and additional processing, particularly when using convolutional neural networks (CNN). A more effective multiresolution method to express an image’s texture is the GW transform [57]. Complex wavelets like the GW have outputs that are made up of both real and imaginary elements. GW is frequently employed in the field of medical image analysis, because it may yield discriminative information in various directions and scales. By convolving the Gabor kernels with the image, GW subbands are produced representing combinations of scales and orientations. In this study, fundus images are analyzed using GW with two scales and orientations. The output of this analysis is four subbands corresponding to different scale-orientation blend. This study uses images of Gabor magnitude and plots the coefficient of each of the subands, and then converts id into image. Next, these images are used to train the CNNs in a later stage of GabROP. Two scales (0.176, 0.25) and two orientations (0, 90) are used in the GW analysis. The primary benefit of GW is that they support multiresolution analysis (analysis at different resolutions or scales and orientations). Besides that, wavelets permit simultaneous decimation in space and frequency, whereas previous transforms enable frequency decimation in the forward transform and in time (or position) in the inverse transform. This dual information will allow the DL models to provide greater insight and details regarding the representation of input data, thus improving the diagnostic performance [58,59]. The result of GW analysis is generating four images (GW-1, GW-2, GW-3, GW-4) representing the magnitude of GW. Samples of the generated GW images for both classes of the ROP dataset are shown in Figure 3. Note that the GW was implemented using the reference [60].

After generating the sets of GW images, these images along with the original fundus images are resized to be equal to 224 × 224 × 3, 224 × 224 × 3, and 256 × 256 × 3 corresponding to the dimension of the input layers of MobileNet, ResNet-50, and DarkNet-53, respectively. We subsequently employed data augmentation to enhance the amount of available photos in order to improve the quality of the training dataset, as explained by [61]. The same augmentation methods used in [46] were employed in this study.

#### 3.2.2. Multiple CNN Training and Extraction of Features

This stage represents the use of TL to modify three pre-trained CNNs that were earlier trained from huge data such as ImageNet in order to identify ROP eye disease. The three CNNs include ResNet-50, MobileNet, and DarkNet-53. To be able to use these CNNs as feature extracted, there are some procedures need to be done. First, the fully connected (FC) layers of each CNN are adjusted to two to be equal to the number of labels in the ROP dataset used in this article rather than the 1000 labels of ImageNet. Then, these three modified pre-trained CNNs are retrained with the ROP dataset. Furthermore, some of their parameters (described later in the parameter setup section) are modified. Each of the three CNNs is initially retrained with the virgin fundus images. When the re-training procedure is ended, TL is further employed to extract spatial deep features from a certain layer of each CNN. These layers are “pool 5” for MobileNet, “avg_pool” for ResNet-50, and “avg 1” for DarkNet-50. The dimensions of these features are 2048, 1280, and 1024 for ResNet-50, MobileNet, and DarkNet-53 respectively. Likewise, the same pre-trained CNNs are individually retrained with each set of the four subbands of the GW images. Afterward, textural features are extracted from the same pooling layers of each CNN trained with each set of GW images, resulting in 2048, 1280, and 1024 textural features for ResNet-50, MobileNet, and DarkNet-53, respectively.

#### 3.2.3. Triple Fusion

There are three fusion stages in GabROP. The DWT which offers spectral-temporal information is initially utilized to combine the textural features generated from the CNNs trained using textural GW images. The selection of DWT was made for the simple reason that it is a well-known technique and is frequently employed, mostly in the medical field. DWT can show temporal-frequency representations from any set of data and decrease the data’s enormous dimensions. To analyze data, DWT employs a class of perpendicular basis functions known as wavelets [62]. The DWT procedure is carried out by passing the data through low and high pass filters in the case of 1-D data, such as the deep spatial features extracted from the three CNNs used in GabROP. The data dimension is then decreased by a downsampling procedure. Following this phase, the approximation coefficients CA1 and detail coefficients CD1 will be formed, making a total of two sets of coefficients. This procedure can be repeated for the approximation coefficients CA1 to achieve a second level of decomposition, and once more two sets of coefficients will be created: the second-level approximation coefficients CA2 and detail coefficients CD2. To build DWT with numerous decomposition levels, this procedure can be repeated. In this study, the four sets of features extracted from each CNN trained separately with each set of GW images are concatenated and fed to DWT to fuse these features and generate a reduced set of features demonstrating textural-spectral-temporal representation of the ROP disease. Four levels of DWT decompositions are employed for the procedure. This process is carried out to evaluate the benefits of DL and textural-based feature extraction methods. The mother wavelet is the Haar wavelet. The fourth detailed coefficients are used in this step in order to reduce the dimension of features generated after fusion. The length of features after the DWT process is 512, 320, and 256 for ResNet-50, MobileNet, and DarkNet-53 respectively.

After that, in the second step of the fusion process, spatial information taken from each CNN trained with the original photos is concatenated with textural-spectral-temporal data taken from CNNs trained using textural GW sets of images (fourth detailed coefficients of DWT). In the end, DCT are fed with the concatenated features of the three CNNs that were trained independently either with the virgin data or the sets of GW images. DCT is used in this step to further reduce the dimension of features resulting from the third fusion procedure. To separate data into their fundamental frequency components, DCT is widely utilized. Information is represented as a sum of varying-frequency cosine functions. The data are typically transformed using the DCT into coefficients, which are divided into two categories: low frequencies are known as (DC coefficients) and high frequencies are known as (AC coefficients). Signals at a high-frequency show noise and little fluctuations (details). Low frequencies are correlated with light surroundings. The dimensions of the DCT coefficient matrix coincide with the input data [63]. The DCT does not function as a reduction step by itself. The vast majority of the important details from the input can be compressed into a more compact set of coefficients by carrying out a second reduction phase in which a small number of coefficients are selected making feature vectors. Zigzag scanning can be employed later to choose a smaller set of DCT coefficients. An ablation study is conducted to investigate the impact of varying the number of DCT coefficients on the classification accuracy.

#### 3.2.4. ROP Diagnosis

GabROP’s diagnosis phase is used to recognize ROP and categorize photos as either having or not having the illness. Three well-known machine learning classifiers are used for this classification: SVM, linear discriminant analysis (LDA), and ensemble subspace discriminate (ESD). The classification application of Matlab 2020 is used to build these classifiers. Using a 5-fold cross-validation procedure, the results are validated. The kernel function used for SVM is linear, and the number of discriminate is 100 for the ESD classifier.

## 4. Performance Evaluation and Setup

Some parameters are tweaked while others are left unchanged when training the three CNNs. The minibatch size is set to 10, the learning rate is set to 0.0003, and the number of epochs is set to 10. These are the hyper-parameters changed in the CNNs. To calculate the training performance one time per epoch, a validation frequency of 1246 was selected. Stochastic gradient descent with momentum was used as the learning approach to develop the three CNNs. Data that were qualified for building DL models as described in [56] are used to construct GabROP where set 1 is employed to avoid imbalance problem occurring in set 2. Sensitivity, F1-score, accuracy, precision, and specificity are the evaluation measures used to analyze GabROP’s performance. Furthermore, the confusion matrix, receiving operating characteristics (ROC), and AUC are employed. The following mathematical functions are used to calculate these measures.

(1) $$Accuracy=\frac{TP+TN}{TN+FP+FN+TP}\;$$

(2) $$Sensitivity=\frac{TP}{TP+FN}$$

(3) $$Specificity=\frac{TN}{TN+FP}$$

(4) $$Precision=\frac{TP}{TP+FP}$$

(5) $$F1-Score=\frac{2\times TP}{2\times TP+FP+FN}$$

where the total number of photos that were accurately identified as diseased is known as true positives (*TP*). The total number of photos that were correctly identified as being disease-free is known as true negative (*TN*). The total number of photos that were misdiagnosed as having an illness is known as a false-positive (*FP*) count. The total of photos that were inaccurately classified as being disease-free is known as a false-negative (*FN*).

## 5. Results

This section presents the GabROP’s triple fusion stage results. First, in the initial fusion stage, DWT is employed to fuse the textural information provided by the textural features that were learned by CNNs utilizing textural GW sets of images. After that, in the second step of the fusion process, spatial information extracted from each CNN trained with the original fundus photos is combined with textural-spectral-temporal features obtained from the CNNs trained on textural GW images. In the end, DCT is utilized to further merge the three CNNs’ fused features.

### 5.1. First Fusion Stage Results

**Figure 4:**
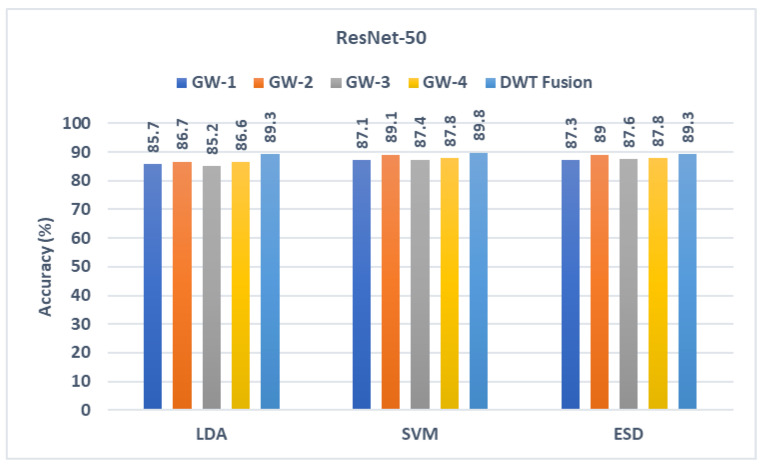
Diagnostic accuracy of the three classifiers trained with the features extracted from ResNet-50 learned using the individual GW images compared the fused DWT features.

**Figure 5:**
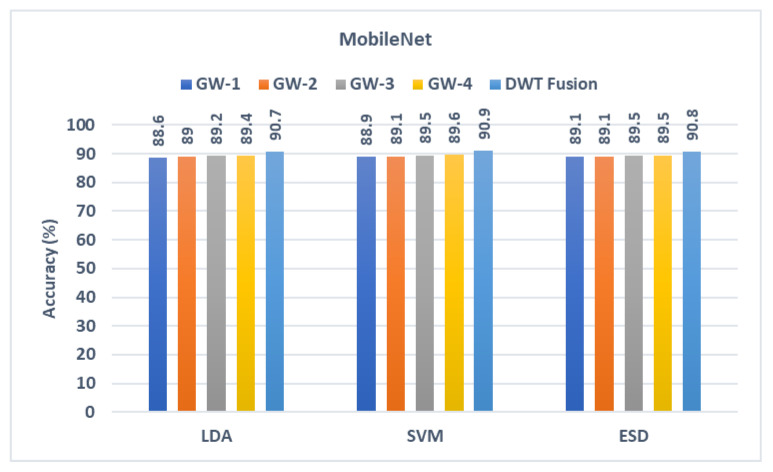
Diagnostic accuracy of the three classifiers trained with the features extracted from DarkNet-53 learned using the individual GW images compared to the fused DWT features.

**Figure 6:**
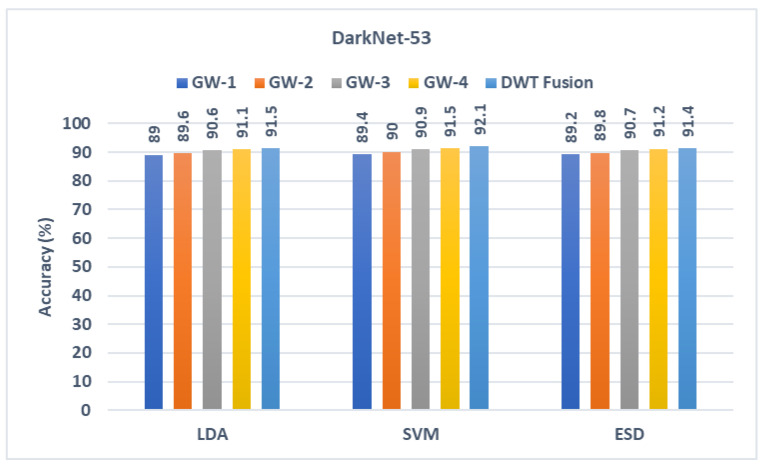
Diagnostic accuracy of the three classifiers trained with the features extracted from MobileNet learned using the individual GW images compared to the fused DWT features.

The outputs from the three classifiers built with texture features acquired from the three CNNs learned using the four sets of GW photos are presented in this section. These results are compared with the output of the classifiers trained with the fused textural-spectral-temporal features obtained after the DWT process (first fusion stage). These comparisons are shown in Figure 4, Figure 5 and Figure 6. It can be noted from Figure 5 that the fusion step is done using DWT has improved the performance of the three classifiers. This is because diagnostic accuracy after fusing textural-spectral-temporal features obtained via the DWT fusion step is 89.3%, 89.8%, and 89.3% for LDA, SVM, and ESD classifiers, respectively. These results are higher than those obtained using the individual texture features extracted from the ResNet-50 learned independently with each GW set of images. Note that the DWT fusion not only enhanced the performance of GabROP, but also successfully reduced the number of features used to construct the classifiers from 2048 to 512 in the case of ResNet-50.

Likewise, the diagnostic accuracies of the three classifiers have improved after the DWT fusion step. This is clear as the accuracy in case textural-spectral-temporal features of DarkNet-50 reached 91.5%, 92.1%, and 91.5% for LDA, SVM, and ESD classifiers, respectively. Similarly, the accuracy of MobileNet’s textural-spectral-temporal features is 90.7%, 90.9%, and 90.8% for LDA, SVM, and ESD classifiers. These enhanced accuracies are achieved with a reduced set of features having a size of 320 and 256 instead of 1280 and 1024 for DarkNet-53 and MobileNet.

### 5.2. Second Fusion Stage Results

**Figure 7:**
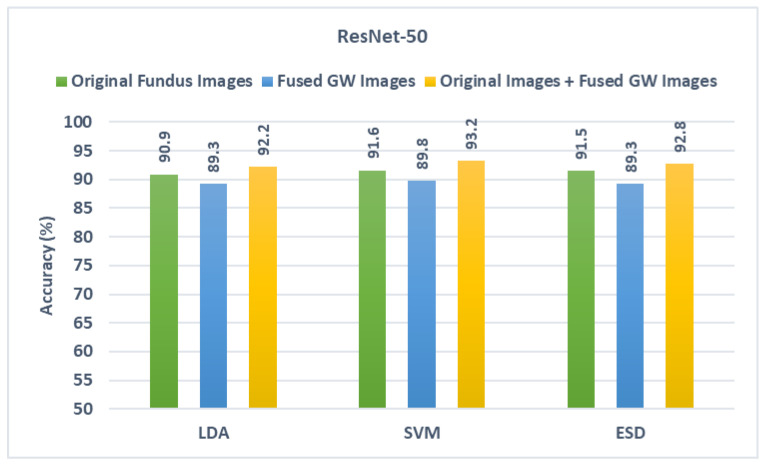
Diagnostic accuracy of the three classifiers trained with spatial features extracted from ResNet-50 learned using the original fundus images compared the fused DWT features obtained by CNNs learned with GW images and the combination of the two.

**Figure 8:**
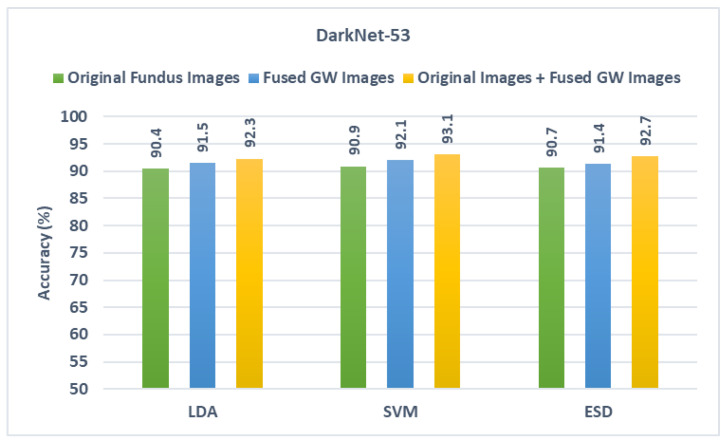
Diagnostic accuracy of the three classifiers trained with spatial features extracted from DarkNet-53 learned using the original fundus images compared the fused DWT features obtained by CNNs learned with GW images and the combination of the two.

**Figure 9:**
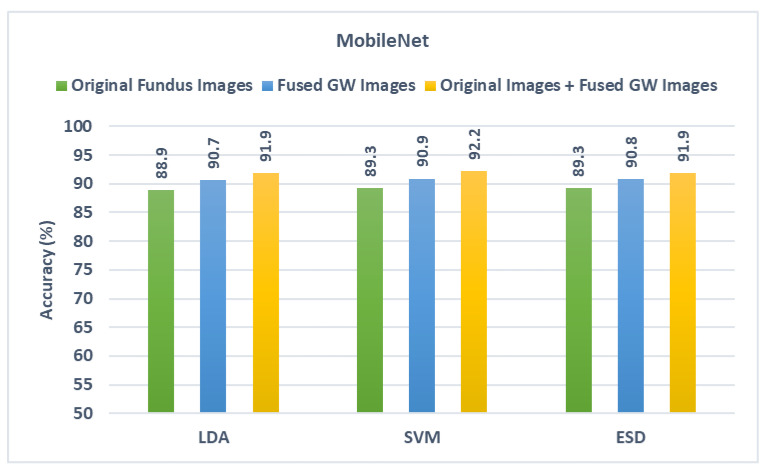
Diagnostic accuracy of the three classifiers trained with spatial features extracted from MobileNet learned using the original fundus images compared the fused DWT features obtained by CNNs learned with GW images and the combination of the two.

This stage depicts the fusion of spatial deep features obtained from the CNNs after they were trained using the actual fundus pictures and those combined texture-spectral-temporal features generated in the first fusion stage (combining textural features taken from each CNN that was trained with different GW pictures). The results of the fusion stage are compared with the results obtained when feeding the classifiers with spatial deep features mined via the CNNs fed with the actual fundus pictures. These results are presented in this section and displayed in Figure 7, Figure 8 and Figure 9. It can be observed from Figure 7 that for ResNet-50, the combination of spatial deep features with textural-spectral-temporal features obtained through training the CNNs with GW images (second fusion stage) is capable of further boosting the performance of GabROP. This means that merging texture information obtained through GW textural analysis method with spatial information attained using the original fundus images is preferable to using only the actual fundus images to feed DL models. Evidently, given the accuracy following the second fusion step is 92.2%, 93.2%, and 92.8% for LDA, SVM, and ESD, respectively, these accuracy levels surpass those attained by the same classifiers built using the spatial deep features of the original fundus images and the combined texture-spectral-temporal features of GW images.

Likewise, for DarkNet-53 and MobileNet results shown in Figure 8 and Figure 9, the same scenario is noticed as the diagnostic accuracy achieved after the second fusion stage is higher than that obtained in the first fusion stage. The accuracies attained using the three classifiers after the second fusion stage are also greater than those reached using the spatial deep features of the original fundus images. This is obvious as the accuracy achieved after the second fusion stage in the case of DarkNet-53 is 92.2%, 93.2%, and 92.7% and in the case of MobileNet is 91.9%,92.2%, and 91.9% for the LDA, SVM, and ESD classifiers, respectively. These results that using textural images of GW along with the original fundus images is superior to utilizing only the original fundus images in diagnosing ROP.

### 5.3. Third Fusion Stage Results

**Figure 10:**
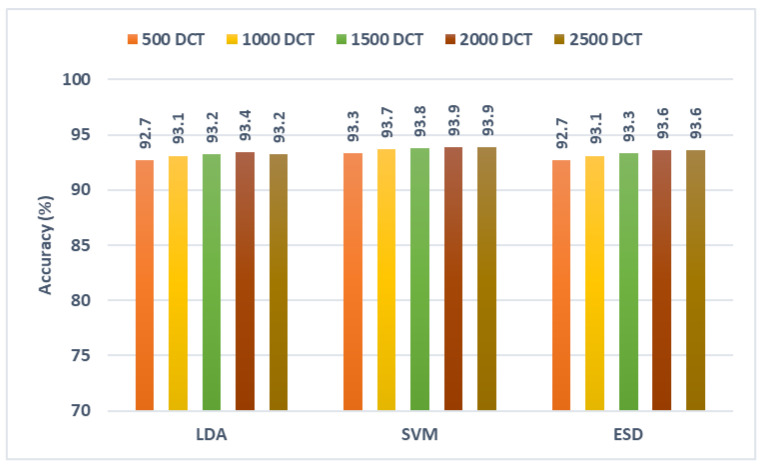
Diagnostic accuracy of the three classifiers trained with integrated features of the third fusion stage (fusing of the second fusion stage features of the three CNNs using DCT) versus the number of DCT features.

In this section, the results of the third fusion stage are discussed. This fusion stage represents the integration of the fused features of the previous stage of the three CNNs using DCT. An ablation study is made to show the change in diagnostic accuracy with the change in the number of DCT coefficients selected after the third fusion stage. The results of the ablation study are displayed in Figure 10. This figure shows that for the LDA classifier, the peak accuracy of 93.4% is attained using 2000 DCT coefficients. Similarly, for the SVM classifier, the highest diagnostic accuracy of 93.9% is attained using 2000 DCT coefficients. Likewise, in the case of the ESD classifier, the maximum diagnostic accuracy of 93.6% is attained with 2000 DCT coefficients. These accuracies are greater than that obtained in the second fusion stage which confirms that merging spatial features with textural-spectral-temporal features extracted from distinct CNNs could boost the diagnostic performance of ROP diagnosis.

**Figure 11:**
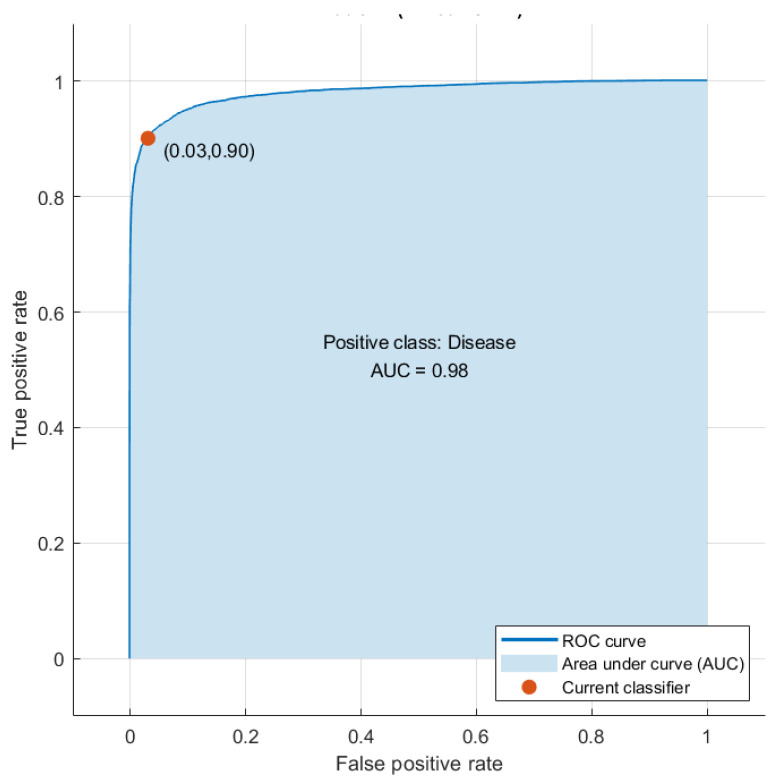
ROC curve and the AUC of the SVM classifier trained with integrated features of the third fusion stage (fusing of the second fusion stage features of the three CNNs using DCT (2000 features)).

Some other performance measures are also calculated to access the performance of the three classifiers of GabROP, including precision, sensitivity, F1-score, and specificity, using the 2000 DCT features. Table 2 presents these metrics, revealing that the F1-scores for the LDA, SVM, and ESD classifiers are 92.52%, 93.02%, and 92.69%, respectively, with precision of 95.57%, 96.11%, and 96.01%, specificity of 96.54%, 96.96%, and 96.90%, and sensitivity of 89.65%, 90.12%, and 89.59%. A diagnostic tool must achieve accuracy and specificity better than 95% and a sensitivity larger than 80% in order to be considered effective, as stated in [64,65]. Based on the performance metrics shown in Table 2, GabROP is therefore recognized as a useful CAD system that may be applied to automatically identify ROP. Figure 11 shows the ROC curve and AUC for SVM classifier trained with the integrated features of the third fusion stage (fusing of the second fusion stage features of the three CNNs using DCT (2000 features)). The figure shows that the AUC is equal to 0.98.

## 6. Discussion

Premature infants weighing less than 2000 g are frequently affected with ROP, a vascular disorder. It might result in blindness or other visual impairment. Every year, the number of ROP cases increases globally. Because the disease develops at an unacceptably quick pace, the time allotted for treatment is limited. Therefore, proper and timely ROP diagnosis and treatment are essential to preventing the disease’s rapid progression, blindness, visual impairment, and other adverse effects. Maintaining a favorable prognosis and regular visual function are also crucial. ROP’s imaging modalities and diagnostic procedures are challenging and face a number of obstacles, such as a shortage of qualified ophthalmologists, particularly in poor nations. However, early detection is now achievable thanks to the most recent innovations in AI and screening techniques such as Retcam. Automatic and precise diagnosis of ROP in its early phases can be provided by diagnostic tools based on DL methods, eliminating its drawbacks and limits while also cutting down on the time and labor required for manual diagnosis.

**Figure 12:**
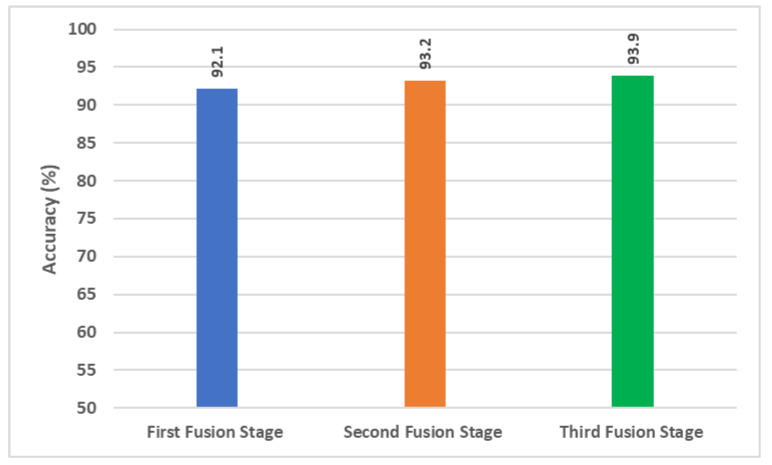
Comparison among the highest accuracy attained in each fusion stage of GabROP.

To achieve an accurate and automatic diagnosis, this study introduced an effective tool namely GabROP based on three DL techniques. Textural analysis methods such as GW have shown their great ability to obtain textural information that aid AI in the accurate diagnosis of several diseases. Thus, GabROP employed GW for analyzing fundus images of the ROP dataset. All previous studies used the original fundus images to build their diagnostic models. However, in this study, GW was used to produce multiple sets of GW images that were utilized to train DL models along with the original fundus images. GabROP consists of three fusion stages. In the former fusion stage, features extracted from each DL model trained with each GW set of images are fused using DWT which consequently results in textural-spectral-temporal representation. Next, in the following stage, spatial features obtained from each CNN constructed with the original fundus images are concatenated with the fused features of the first stage of fusion. Lastly, integrated features of each CNN attained from the second fusion stage were merged using DCT. Figure 12 illustrates a comparison between the highest accuracy achieved in each fusion stage.

Figure 12 indicates that for each CNN, fusing texture features obtained from multiple GW images using DWT is better than using features attained with one GW. Furthermore, in some cases, e.g., Dark-53 and MobileNet, merging features obtained from each of these CNN learned with multiple sets of GW images is superior to utilizing features attained through training these CNNs with the original fundus images (as shown in Figure 8 and Figure 9). In other words, training CNNs with multiple sets of GW images may be more significant and useful than using the original fundus images. The maximum accuracy attained (93.2%) in the second fusion stage is higher than that reached in stage 1 (92.1%). This proves that integrating spatial features extracted from every CNN with textural-spectral-temporal features attained through training these CNNs with multiple sets of GW images could boost GabROP performance. Finally, merging features attained in the second stage of fusion of the three CNN is capable of a further enhancement in the diagnostic performance of GabROP. This is because the accuracy achieved in the third fusion (93.9%) is higher than that of the second and first fusion stages. This verifies that combining features of multiple CNNs having distinct architectures is more beneficial than using only one structure.

### 6.1. Comparisons

The lack of ROP datasets is the main barrier to this study’s success. Since all datasets utilized in the literature to our knowledge are private, it was challenging to assess how well GabROP performed in comparison to similar earlier diagnostic methods. To our knowledge, the two articles [45,55] are the only ones that used the same dataset that was employed to build GabROP. In [56], the authors built their diagnostic tool using three CNNs individually. Moreover, both studies [45,55] employed the original fundus images to construct their diagnostic models. Alternatively, GabROP used GW to analyze fundus images and generate multiple sets of GW images to train DL models along with the original fundus images. Furthermore, instead of utilizing a single CNN, GabROP combined the textural-spectral-temporal features of three CNNs. Table 3 compares the performance of GabROP with that of the CNNs, namely AlexNet, GoogleNet, and VGG-16. Other end-to-end models that were identified in the literature were also compared to it. The accuracy achieved by GabROP (93.9%) is higher than that obtained by the DIAROP tool (93.2%) [46]; 77.9% of AlexNet, 73.9% of GoogleNet, and 80.4% of VGG-16 employed in [56], demonstrating the method’s superiority over the others. The accuracy attained with GabROP is also higher than the 86.5%, 87.9%,90.9%, 91.42%, 91%, and 91.48% achieved using Xception, MobileNet, InceptionResNet, Inception, Dark-53, and ResNet-50 CNNs. This indicates that GabROP outperforms end-to-end CNNs in terms of accuracy. Due to GabROP’s superior performance, ophthalmologists can utilize it to make a more accurate diagnosis of the ROP. It is an effective automated model that can shorten examination times and ease the strain on ophthalmologists during the diagnostic process.

### 6.2. Limitations and Future Directions

Despite the encouraging outcomes, this study had a number of shortcomings. The true diagnosis of ROP includes ROP phases and zones that were not covered in the research, as well as disease ROP, acute ROP, aggressive posterior ROP, and pre-threshold ROP. Additionally, it did not take into account classifying the severity of the ROP disease. Furthermore, the absence of segmentation techniques is one of GabROP’s shortcomings. Additionally, the optimization techniques for choosing deep learning hyperparameters were not investigated. These constraints will be covered in upcoming research. More CNNs may be used in future research. Further, optimization techniques will be studied to select optimization parameters. Moreover, DL-based methods, such as U-Nets and their variants, will be employed to segment retinal characteristics, including the optic disc, the vessel, the demarcation line, and the ridge, in order to better describe the attributes of ROP. In addition, forthcoming research will use GabROP to determine the severity of the ROP condition.

## 7. Conclusions

This work developed an effective and automatic CAD tool called GabROP based on a combination of DL approaches to identify ROP disease. Instead of utilizing only the original fundus image to perform ROP diagnosis, GabROP employed GW to analyze these images and produce multiple sets of GW images. These generated images were used along with the original fundus images to train three DL models. GabROP is made up of four stages: GW image generation and preprocessing, multiple CNN training and extraction of features, triple fusion, and diagnosis stages. The fusion step was conducted in three contexts. In the initial stage of the fusion process, DWT was used to fuse the features obtained from each DL model trained with each set of GW images, representing the textural-spectral-temporal demonstration. The spatial features obtained from each CNN built using the original fundus pictures were then concatenated with the fused features of the first phase of fusion in the following stage. Finally, DCT was used to combine the incorporated features of each CNN obtained from the second stage of fusion. The performance of the first fusion stage indicated that training DL models with multiple sets of GW images are preferred over using a single set of GW images. Furthermore, using multiple sets of GW images may improve diagnostic accuracy compared to using the fundus images alone. The results obtained from second stage of fusion demonstrated that combining spatial data obtained from the original fundus images with temporal-spectral-textural information is preferable to using the latter alone. Additionally, the third fusion results demonstrated that the combined textural-spectral-temporal features of several CNNs might improve the diagnosis of ROP condition. The effectiveness of GabROP was confirmed through comparisons with end-to-end DL CNNs and recent comparable CAD tools. Even without using any segmentation or detection DL models, GabROP achieves a comparable AUC of 0.98 which is close to that attained by the classification task of the ADAM challenge. Future directions will take into consideration applying DL models for segmenting and detecting ROP and optic disc from fundus images prior to classification. GabROP is regarded as a strong and effective CAD tool that can accurately and automatically diagnose ROP disease. The manual effort required for the diagnosis process and the amount of time spent on examination can both be decreased using GabROP.

## Figures and Tables

**Table 1:** Characteristics of the subjects.

Attribute	Set 1	Set 2
**Gender**		
Female	7726	754
Male	10,075	988
**Age**	31.9 (24–36.4)	32.0 (25–36.2)
**Birth Weight**	1.49 (0.63–2.00)	1.50 (0.78–2.00)
**Classification of Images**		
ROP Images	8090	155
Not ROP Images	9711	1587

**Table 2:** The efficiency indicators (%) attained with the three GabROP classifiers using the 2000 DCT features.

Classifier	Precision	Specificity	Sensitivity	F1-score
**LDA**	95.57	96.54	89.65	92.52
**SVM**	96.11	96.96	90.12	93.02
**ESD**	96.01	96.90	89.59	92.69

**Table 3:** An evaluation of GabROP in contrast to recent ROP studies using the same dataset.

Article	Model	Accuracy (%)
[56]	**GoogleNet**	73.9
[56]	**AlexNet**	77.9
[56]	**VGG-16**	80.4
[46]	**ResNet + Inception + InceptionResNet**	93.2
[48]	**Inception-ResNet V2**	90.9
[48]	**Xception**	86.95
[44]	**ResNet-50**	91.48
[47]	**Inception V3**	91.42
[66]	**MobileNet**	87.94
[67]	**DarkNet-53**	91.0
	**GabROP**	93.9%

## Data Availability

The dataset utilized in this article is available through the IEEE Dataport. https://ieee-dataport.org/documents/dnn-classifier-wide-angle-retinal-images-computer-aided-screening-rop, accessed on 12 October 2022. Codes will be available upon request.
